# The relationship of insulin resistance and diabetes to tau PET SUVR in middle-aged to older adults

**DOI:** 10.1186/s13195-023-01180-2

**Published:** 2023-03-17

**Authors:** Gilda E. Ennis, Tobey J. Betthauser, Rebecca Langhough Koscik, Nathaniel A. Chin, Bradley T. Christian, Sanjay Asthana, Sterling C. Johnson, Barbara B. Bendlin

**Affiliations:** 1 Wisconsin Alzheimer’s Disease Research Center, University of Wisconsin-Madison School of Medicine and Public Health, Madison, WI USA; 2 Division of Geriatrics and Gerontology, Department of Medicine, University of Wisconsin-Madison School of Medicine and Public Health, Madison, WI USA; 3 Wisconsin Alzheimer’s Institute, University of Wisconsin-Madison School of Medicine and Public Health, Madison, WI USA; 4 Waisman Laboratory for Brain Imaging and Behavior, University of Wisconsin-Madison, Madison, WI USA; 5 Department of Medical Physics, University of Wisconsin-Madison, Madison, WI USA; 6 Geriatric Research Education and Clinical Center, William S. Middleton Hospital Department of Veterans Affairs, Madison, WI USA

**Keywords:** Insulin resistance, Type 2 diabetes, Tau PET, Amyloid PET, Preclinical Alzheimer’s disease

## Abstract

**Background:**

Insulin resistance (IR) and type 2 diabetes have been found to increase the risk for Alzheimer’s clinical syndrome in epidemiologic studies but have not been associated with tau tangles in neuropathological research and have been inconsistently associated with cerebrospinal fluid P-tau181. IR and type 2 diabetes are well-recognized vascular risk factors. Some studies suggest that cardiovascular risk may act synergistically with cortical amyloid to increase tau measured using tau PET. Utilizing data from largely nondemented middle-aged and older adult cohorts enriched for AD risk, we investigated the association of IR and diabetes to tau PET and whether amyloid moderated those relationships.

**Methods:**

Participants were enrolled in either the Wisconsin Registry for Alzheimer’s Prevention (WRAP) or Wisconsin Alzheimer’s Disease Research Center (WI-ADRC) Clinical Core. Two partially overlapping samples were studied: a sample characterized using HOMA-IR (*n*=280 WRAP participants) and a sample characterized on diabetic status (*n*=285 WRAP and *n*=109 WI-ADRC). IR was measured using the homeostasis model assessment of insulin resistance (HOMA-IR). Tau PET employing the radioligand ^18^F-MK-6240 was used to detect AD-specific aggregated tau. Linear regression tested the relationship of IR and diabetic status to tau PET standardized uptake value ratio (SUVR) within the entorhinal cortex and whether relationships were moderated by amyloid assessed by amyloid PET distribution volume ratio (DVR) and amyloid PET positivity status.

**Results:**

Neither HOMA-IR nor diabetic status was significantly associated with tau PET SUVR. The relationship between IR and tau PET SUVR was not moderated by amyloid PET DVR or positivity status. The association between diabetic status and tau PET SUVR was not significantly moderated by amyloid PET DVR but was significantly moderated by amyloid PET positivity status. Among the amyloid PET-positive participants, the estimated marginal tau PET SUVR mean was higher in the diabetic (*n*=6) relative to the nondiabetic group (*n*=88).

**Conclusion:**

Findings indicate that IR may not be related to tau in generally healthy middle-aged and older adults who are in the early stages of the AD clinicopathologic continuum but suggest the need for additional research to investigate whether a synergistic relationship between type 2 diabetes and amyloid is associated with increased tau levels.

**Supplementary Information:**

The online version contains supplementary material available at 10.1186/s13195-023-01180-2.

## Background

Insulin resistance (IR), a condition that disrupts metabolic homeostasis and increases the risk for type 2 diabetes [1, 2], has been associated with an increased risk for Alzheimer’s clinical syndrome [3, 4]. IR within the peripheral tissues has been hypothesized by some to alter neuronal insulin signaling and contribute to the development of neurofibrillary tangles (NFT) [5], one of the pathologic hallmarks of Alzheimer’s disease (AD). Some studies demonstrate that animals fed high-fat diets to model IR have higher levels of soluble hyperphosphorylated tau [6–8]. Tau hyperphosphorylation is thought to precede aggregation of tau in humans into the insoluble paired helical filaments (PHF) [9–11] that comprise the NFTs of AD [12]. In some human studies [13, 14], IR has been related to higher levels of a soluble form of tau phosphorylated at threonine 181 (P-tau181), an epitope-specific for AD [15]. Higher P-tau181 in the cerebrospinal fluid (CSF) has been associated with higher homeostatic model assessment of insulin resistance (HOMA-IR) values in cognitively unimpaired older adults [13] and in cognitively unimpaired middle-aged and older adults who are carriers of the *APOE* ε4 allele [14]. However, significant associations between IR and CSF P-tau181 have not been consistently found [16], and increased tau phosphorylation has not been consistently isolated in animal models of IR [17]. Similarly, type 2 diabetes, which is characterized by IR as well as insulin deficiency [2, 18], has been shown to associate with higher CSF P-tau181 in some studies [19] but not in others [20].

Although elevations in CSF P-tau181 have been found to predict the later development of NFTs [21], P-tau may not be a direct marker of NFTs or PHFs like tau positron emission tomography (PET) [22, 23]. Instead, higher concentrations of CSF P-tau181, at least in early AD, may represent a neuronal response to amyloid exposure [21, 22, 24]. In the few neuropathological studies that have investigated the relationship between antemortem IR and NFTs, IR has not been significantly related to regional NFT spread assessed by the Braak score [25, 26]. Similarly, type 2 diabetes has not been related to either the presence or quantity of NFTs in post-mortem studies [27–30]. Approximately one-half of the cases in these studies had an antemortem diagnosis of dementia [26, 30], suggesting that results may be more relevant to a later stage of the AD continuum. Whether IR and diabetes are related to NFTs in adults without dementia who are in an earlier stage of AD, when treatment is more likely to be effective, has not been well-studied.

In addition to their effects upon metabolism, IR and type 2 diabetes also impact the vasculature [31, 32] and increase the risk for cardiovascular disease (CVD) [33, 34]. Some research suggests that cardiovascular disease risk and vascular dysfunction are related to greater tau burden in individuals with higher cortical amyloid [35, 36]. Rabin et al. (2019) found that in a sample of cognitively unimpaired older adults, participants with both higher Framingham Heart Study (FHS) CVD risk and higher amyloid PET distribution volume ratio (DVR) had greater tau PET standardized uptake value ratio (SUVR). However, another similar study using the same PET tracers in cognitively unimpaired adults did not find that an interaction between FHS CVD risk and amyloid was related to tau [37]. That study had fewer participants who were *APOE4* allele carriers and a smaller range of CVD risk, which could have influenced results. Neither study specifically examined the association of IR and amyloid on tau PET SUVR.

We tested the association of IR and diabetic status to aggregated tau, using MK-6240 PET, in a sample enriched for AD risk due to an increased proportion of *APOE* ε4 allele carriers and comprised largely of middle-aged and older adults who were cognitively unimpaired. We also explored whether the relationship of IR and diabetic status to aggregated tau was moderated by amyloid burden, assessed using PiB PET.

## Methods

### Participants

Participants were enrolled in either the Wisconsin Registry for Alzheimer’s Prevention (WRAP), a longitudinal study of middle-aged and older adults enriched for AD risk [38], or the Wisconsin Alzheimer’s Disease Research Center (WI-ADRC) Clinical Core. Diagnosis of mild cognitive impairment and dementia was determined by a multidisciplinary consensus review team [38, 39]. Relevant medical and cognitive data were evaluated to determine cognitive status based upon NIA-AA criteria [40, 41]. All participants provided written informed consent prior to study participation. Study procedures were approved by the University of Wisconsin – Madison Institutional Review Board.

Participants were selected depending upon the availability of tau and amyloid PET, diabetic status data, and fasting glucose and fasting insulin, which are required to calculate HOMA-IR [42] Because WRAP and not WI-ADRC participants have insulin collected at regular study visits, only WRAP participants were included in the sample (i.e., the “HOMA-IR sample”) utilized to test the relationship between IR and tau PET SUVR. *N*=281 WRAP participants had PET as well as fasting glucose and insulin for inclusion in the HOMA-IR sample. Because HOMA-IR is not recommended as a measure for IR in people on insulin therapy [42], one participant with type 2 diabetes who was receiving insulin therapy was excluded, resulting in *N*=280 participants in the HOMA-IR sample. N=394 participants (*n*=285 WRAP [which included *n*=280 from the HOMA-IR sample] and *n*=109 WI-ADRC) had PET and diabetic status data; therefore, they were included in the sample used to test the relationship between diabetic status and tau PET SUVR (i.e., the “Diabetic Status sample”). Descriptive statistics of demographic characteristics and study variables for both samples can be found in Tables 1 and 2.

**Table 1:** Descriptive statistics of demographic and health characteristics and study variables. Data presented are means (standard deviations) or counts (%). Between-group differences tested using independent samples *t*-test for continuous variables and Pearson’s chi-square, Fisher’s Exact test, or Fisher-Freeman-Hamilton Exact test for categorical variables^a^

	HOMA-IR sample (*N*=280)	Amyloid PET positive^b^ (*n*=67)	Amyloid PET negative (*n*=213)	*p*	Diabetic Status sample (*N*=394)	Diabetic^c^ (*n*=37)	Non-diabetic (*n*=357)	*p*
Age (years)	68.1 (6.6)	70.8 (4.9)	67.3 (6.8)	<.001	68.0 (7.1)	69.8 (7.4)	67.8 (7.1)	.11
Sex (female)	186 (66.4)	41 (61.2)	145 (68.1)	.30	261 (66.2)	24 (64.9)	237 (66.4)	.85
Race/ethnic group:				1.0				<.001
White	263 (93.9)	63 (94.0)	200 (93.9)		358 (90.9)	24 (64.9)	334 (93.6)	<.001
Black	12 (4.3)	3 (4.5)	9 (4.2)		22 (5.6)	9 (24.3)	13 (3.6)	<.001
American Indian, Asian, Hispanic/Spanish^d^	5 (1.8)	1 (1.5)	4 (1.9)		14 (3.6)	4 (10.8)	10 (2.8)	.01
Education (years)	16.2 (2.2)	16.1 (2.2)	16.2 (2.2)	.75	16.1 (2.4)	15.6 (2.3)	16.2 (2.4)	.19
					***n*=272** HOMA2-IR	***n*=22**	***n*=250**	
HOMA2-IR^e^	1.1 (0.7)	1.0 (0.4)	1.1 (0.7)	.18	1.1 (0.7)	1.6 (0.8)	1.0 (0.7)	<.001
					***n*=381** Glucose	***n*=32**	***n*=349**	
Glucose (mg/dL)	98.6 (14.7)	96.1 (9.8)	99.4 (15.8)	.12	98.5 (14.1)	128.9 (23.5)	95.7 (8.7)	<.001
Prediabetes^f^	74 (26.4)	21 (31.3)	53 (24.9)	.30	100 (25.9)	--	100 (28.0)	
Diabetes	22 (7.9)	1 (1.5)	21 (9.9)	.03	37 (9.4)	37 (100.0)	--	
Diabetic medications:								
Diabetics	15 (5.4)	0 (0)	15 (7.0)	--	27 (6.9)	27 (73.0)		
Non-diabetics^g^	2 (0.8)	0 (0)	2 (0.9)	--	4 (1.0)	--	4 (1.1)	
	***n*=276** *APOE4* allele status	***n*=66**	***n*=210**		***n*=379** *APOE4* allele status	***n*=33**	***n*=346**	
*APOE4* allele status:				<.001				.09
Non-carrier	170 (61.6)	23 (34.8)	147 (70.0)	<.001	228 (60.2)	26 (78.8)	202 (58.4)	
ε2 ε4	7 (2.5)	1 (1.5)	6 (2.9)	>.05	9 (2.4)	1 (3.0)	8 (2.3)	
ε3 ε4	86 (31.2)	33 (50.0)	53 (25.2)	<.001	120 (31.7)	5 (15.2)	115 (33.2)	
ε4 ε4	13 (4.7)	9 (13.6)	4 (1.9)	<.001	22 (5.8)	1 (3.0)	21 (6.1)	
Amyloid PET DVR^h^	1.16 (0.22)	1.50 (0.23)	1.06 (0.05)	<.001	1.17 (0.23)	1.15 (0.24)	1.17 (0.23)	.58
Amyloid PET positive^b^	67 (23.9)	67 (100.0)	0 (0)	--	94 (23.9)	6 (16.2)	88 (24.6)	.25
Tau PET SUVR, EC^i^	1.09 (0.27)	1.30 (0.41)	1.02 (0.15)	<.001	1.12 (0.32)	1.17 (0.42)	1.12 (.31)	.31
Tau PET positive^j^	38 (13.6)	25 (37.3)	13 (6.1)	<.001	61 (15.5)	9 (24.3)	52 (14.6)	.12
Tau PET SUVR, MTL^k^	0.98 (.22)	1.15 (.34)	0.93 (.12)	<.001	1.00 (.27)	1.03 (.34)	1.00 (.26)	.51
Tau PET SUVR, temporal meta-ROI^l^	1.13 (.25)	1.31 (.43)	1.07 (.11)	<.001	1.15 (.31)	1.17 (.31)	1.15 (.31)	.77
Time to tau PET^m^ (years)	1.06 (1.05)	1.12 (1.10)	1.05 (1.03)	.60	.92 (.94)	.89 (.70)	.93 (.97)	.78
MCI^n^	9 (3.2)	7 (10.4)	2 (0.9)	.001	23 (5.8)	3 (8.1)	20 (5.6)	.47
Dementia^n^	0 (0)	0 (0)	0 (0)	--	6 (1.5)	2 (5.4)	4 (1.1)	.10

*Abbreviations*: *DVR* Distribution volume ratio, *EC* Entorhinal cortex, *HOMA2-IR* Homeostasis model assessment of insulin resistance, *MCI* Mild cognitive impairment, *MTL* Medial temporal lobe, *PET* Positron emission tomography, *ROI*, Region of interest, *SUVR*, Standardized uptake value ratio

^a^For categorical variables with >2 cells, *p*-value for main effect is noted first followed by *p*-values for statistically significant post hoc pairwise comparisons

^b^Amyloid PET positive: average Pittsburgh Compound B (PiB) DVR > 1.19 from 8 bilateral regions at PiB PET closest in time to tau PET

^c^Diabetes identified by clinician or self-report of diabetes or fasting glucose ≥ 126 mg/dL

^d^The 3 race/ethnic groups were combined to maintain anonymity for groups with < 3 individuals

^e^HOMA2-IR has no reference range; a value of 1.0 approximates normal (Wallace, Levy, & Matthews, 2004). HOMA2-IR values in the Diabetic Status sample are from WRAP participants only

^f^Prediabetes identified by fasting glucose ≥ 100 mg/dL (American Diabetes Association, 2010)

^g^Off-label use of metformin in non-diabetics

^h^Value represents average PiB PET DVR across 8 bilateral regions

^i^Value represents average tau PET SUVR from bilateral entorhinal cortex

^j^Tau PET positive: average tau PET SUVR > 1.27 from bilateral entorhinal cortex

^k^Value represents average tau PET SUVR from bilateral entorhinal cortex, hippocampus, and amygdala

^l^Value represents average tau PET SUVR from bilateral parahippocampal gyrus, amygdala, fusiform cortex, and inferior and middle temporal gyrus

^m^Time between predictor (HOMA2-IR or diabetic status) and tau PET

^n^Diagnosed using NIA-AA criteria and consensus conference

**Table 2:** Descriptive statistics of demographic and health characteristics and study variables in the Diabetic Status sample characterized according to amyloid PET positivity status. Data presented are means (standard deviations) or counts (%). Between-group differences tested using *t* test for continuous variables and Pearson’s chi-square, Fisher’s exact test, or Fisher-Freeman-Hamilton exact test for categorical variables^a^

	(*N*=394)	Diabetic^b^ amyloid PET positive^c^ (*n*=6)	Non-diabetic amyloid PET positive^c^ (*n*=88)	*p*	Diabetic^b^ amyloid PET negative (*n*=31)	Non-diabetic amyloid PET negative (*n*=269)	*p*
Age (years)	68.0 (7.1)	75.12 (6.4)	71.3 (5.2)	.09	68.7 (7.2)	66.7 (7.2)	.14
Sex (female)	261 (66.2)	4 (66.7)	55 (62.5)	1.0	20 (64.5)	182 (67.7)	.84
Race/ethnic group:				.002			<.001
White	358 (90.9)	3 (50.0)	84 (95.5)	<.001	21 (67.7)	250 (92.9)	<.001
Black	22 (5.6)	1 (16.7)	3 (3.4)	>.05	8 (25.8)	10 (3.7)	<.001
American Indian, Asian, Hispanic/Spanish^d^	14 (3.6)	2 (33.3)	1 (1.1)	<.001	2 (6.5)	9 (3.3)	>.05
Education (years)	16.1 (2.4)	17.3 (2.1)	16.1 (2.3)	.18	15.3 (2.2)	16.2 (2.4)	.05
	***n*=272** HOMA2-IR	***n*=1**	***n*=66**		***n*=21**	***n*=184**	
HOMA2-IR^e^	1.1 (0.7)	0.5	1.0 (0.4)	--	1.7 (0.9)	1.1 (0.7)	<.001
	***n*=381** Glucose	***n*=5**	***n*=85**		***n*=27**	***n*=264**	
Glucose (mg/dL)	98.5 (14.1)	117.6 (31.7)	96.5 (9.1)	.21	131.0 (21.8)	95.5 (8.6)	<.001
Prediabetes^f^	100 (25.9)	--	28 (32.9)		--	72 (27.3)	
Diabetic medications:							
Diabetics	27 (6.9)	3 (50.0)	--		24 (77.4)	--	
Non-diabetics^g^	4 (1.0)	--	0 (0.0)		--	4 (1.5)	
	***n*=379** *APOE4* allele status	***n*=5**	***n*=83**		***n*=28**	***n*=263**	
*APOE4* allele status:							.14
Non-carrier	228 (60.2)	2 (40.0)	27 (32.5)	1.0	24 (85.7)	175 (66.5)	
ε2 ε4	9 (2.4)	0 (0.0)	1 (1.2)		1 (3.6)	7 (2.7)	
ε3 ε4	120 (31.7)	2 (40.0)	40 (48.2)		3 (10.7)	75 (28.5)	
ε4 ε4	22 (5.8)	1 (20.0)	15 (8.1)		0 (0.0)	6 (2.3)	
Amyloid PET DVR^h^	1.17 (0.23)	1.62 (.31)	1.50 (.23)	.25	1.06 (.05)	1.06 (.05)	.72
Tau PET SUVR, EC^i^	1.12 (0.32)	1.94 (.53)	1.37 (.48)	.006	1.02 (.15)	1.03 (.17)	.83
Tau PET positive^j^	61 (15.5)	6 (100.0)	37 (42.0)	.007	3 (9.7)	15 (5.6)	.41
Tau PET SUVR, MTL^k^	1.00 (.27)	1.62 (.49)	1.21 (.40)	.02	.92 (.11)	.93 (.13)	.52
Tau PET SUVR, temporal meta-ROI^l^	1.15 (.31)	1.70 (.44)	1.37 (.53)	.15	1.06 (.11)	1.08 (.11)	.48
Time to tau PET^m^ (years)	.92 (.94)	.96 (.92)	1.02 (1.05)	.90	.88 (.67)	.90 (.94)	.89
MCI^n^	23 (5.8)	1 (16.7)	15 (17.0)	1.0	2 (6.5)	5 (1.9)	.16
Dementia^n^	6 (1.5)	1 (16.7)	3 (3.4)	.24	1 (3.2)	1 (0.4)	.20

*Abbreviations*: *DVR* Distribution volume ratio, *EC* Entorhinal cortex, *HOMA2-IR* Homeostasis model assessment of insulin resistance, *MCI* Mild cognitive impairment, *MTL* Medial temporal lobe, *PET* Positron emission tomography, *ROI*, Region of interest, *SUVR* Standardized uptake value ratio

^a^For categorical variables with > 2 cells, *p*-value for main effect is noted first followed by *p*-values for statistically significant post hoc pairwise comparisons

^b^Diabetes identified by clinician or self-report of diabetes or fasting glucose ≥ 126 mg/dL

^c^Amyloid PET positive: average Pittsburgh Compound B (PiB) DVR > 1.19 from 8 bilateral regions at PiB PET closest in time to tau PET

^d^The 3 race/ethnic groups were combined to maintain anonymity for groups with < 3 individuals

^e^HOMA2-IR has no reference range; a value of 1.0 approximates normal (Wallace, Levy, & Matthews, 2004). HOMA2-IR values in the Diabetic Status sample are from WRAP participants only

^f^Prediabetes identified by fasting glucose ≥ 100 mg/dL (American Diabetes Association, 2010)

^g^Off-label use of metformin in non-diabetics

^h^Value represents average PiB PET DVR across 8 bilateral regions

^i^Value represents average tau PET SUVR from bilateral entorhinal cortex

^j^Tau PET positive: average tau PET SUVR > 1.27 from bilateral entorhinal cortex

^k^Value represents average tau PET SUVR from bilateral entorhinal cortex, hippocampus, and amygdala

^l^Value represents average tau PET SUVR from bilateral parahippocampal gyrus, amygdala, fusiform cortex, and inferior and middle temporal gyrus

^m^Time between predictor (HOMA2-IR or diabetic status) and tau PET

^n^Diagnosed using NIA-AA criteria and consensus conference

### Procedures

#### General

Participants fasted for a minimum of 8 h prior to having their blood collected during a regular biennial or annual visit. Medical diagnoses (e.g., diabetes) and medication history (e.g., antidiabetic medications) were self-reported through medical history questionnaires and/or clinician interviews as part of the source study evaluations. PET imaging was collected during a regular biennial or annual visit or as part of a PET sub-study using a common acquisition protocol.

#### Insulin resistance (IR)

IR was measured using the “updated” homeostasis model assessment of insulin resistance (HOMA2-IR [42];). HOMA2-IR was calculated by entering fasting glucose and insulin into the HOMA2 calculator version 2.2.3 (University of Oxford). HOMA2 modeling has been shown to correlate strongly with the euglycemic clamp and minimal model methods of whole-body insulin sensitivity [42]. A value of 1.0 is considered normal, but there is no reference range. In the HOMA-IR sample, the average HOMA2-IR in non-diabetic participants was 1.0 (*n*=258, SD = .66, interquartile range = .6 to 1.3, full range = 0.1 to 5.6) and the average value in diabetic participants was 1.6 (*n*=22, SD = 0.8, interquartile range = 1.1 to 2.1, full range = .5 to 3.4). Although we are unaware of published cut-points for HOMA2-IR for the US population, a HOMA1-IR cut-point of 2.7 has been used as a threshold for identifying insulin resistance in US samples of nondiabetic adults [43, 44]. This value was equivalent to a HOMA2-IR value of 1.3 in the HOMA2-IR sample. There where *n*=85 (30.4%) participants with HOMA2-IR ≥ 1.3 in that sample. HOMA2-IR and HOMA1-IR, the original HOMA method for calculating IR, were strongly correlated (*r* = .98, *p* < .001). Fasting glucose and insulin collected closest in time and within the same month as or prior to tau PET were used to calculate HOMA2-IR. On average glucose and insulin were collected 1.06 years prior to tau PET (year of tau PET minus year of blood collection: SD = 1.05, interquartile range = .17 to 1.75, full range = −.07 to 5.68).

#### Diabetic status

Diabetic status was determined by clinician or self-report of diabetes or fasting glucose ≥ 126 mg/dL, a value considered diagnostic for diabetes [45]. *N*=14 WI-ADRC participants had a report of type 2 diabetes determined through clinician interviews. There were no WI-ADRC participants without a clinician report of type 2 diabetes who had a fasting glucose ≥ 126 mg/dL. *N*=20 WRAP participants had a self-report of diabetes, and *n*=3 had a high fasting glucose. WRAP participants did not indicate whether diabetes was type 1 or 2, but no participant self-reported being on insulin therapy in the absence of oral antidiabetic medications. Information collected closest in time and within the same month or prior to tau PET was used to determine diabetic status. On average diabetic status information was collected .92 years prior to tau PET (year of tau PET minus year of diabetic status: SD = .94, interquartile range = .16 to 1.45, full range = −.08 to 5.68).

#### Tau and amyloid PET acquisition and processing

^18^F-MK-6240 and ^11^C-Pittsburgh Compound B (PiB) were used to quantify aggregated tau and cortical β-amyloid, respectively, using previously published methods [46, 47]. PET data were collected using either a Siemens Biograph Horizon PET/CT or Siemens EXACT HR+ tomograph. Dynamic data were acquired for 20 min (5 min × 4 frames) following a 70-min uptake period for MK-6240 and 0–70 min (2 min × 5 frames, 5 min × 12 frames) beginning with tracer injection for PiB. T1-weighted magnetic resonance imaging (MRI) was performed to delineate anatomical regions. MRI and PET image processing and quality control were performed using a pipeline that uses MATLAB (The Mathworks, Inc., Natick, MA) and SPM12 (University College London). Details regarding radioligand synthesis, image acquisition, processing, and analysis of MRI, MK-6240 PET images [46], and PiB PET images [47] have been previously described.

#### Amyloid PET DVR

Cortical PiB DVR (Logan graphical analysis, cerebellum gray matter reference region) was averaged across 8 bilateral regions as previously described [48]. A PiB DVR of 1.19 (equivalent to 20.6 Centiloids) was the cut-point for amyloid PET positivity [49]. We used the amyloid PET scan closest in time to tau PET. On average amyloid PET was performed .02 years (i.e., 7.3 days) prior to tau PET (calculated as year of tau PET minus year of amyloid PET) in both the HOMA-IR (SD = .24; interquartile range = 0 to 0; full range = −.58 to 2.64) and Diabetic Status samples (SD = .25; interquartile range = 0 to 0, full range = -.69 to 2.64.).

#### Tau PET SUVR

Tau burden was ascertained using the average MK-6240 standardized uptake value ratio (SUVR; 70–90 min, inferior cerebellum reference region [46]) from the left and right entorhinal cortex (EC). The EC was selected as the primary region of interest (ROI) because it is one of the first to develop NFTs according to Braak pathological staging [50] and tau tracer uptake in this region has been shown to be associated with cognitive decline in preclinical samples [51, 52]. The cut-point for tau PET positivity was an EC tau PET SUVR of 1.27 [51]. To provide context for results obtained using the EC ROI, we examined secondarily a medial temporal lobe (MTL) composite (bilateral entorhinal cortex, hippocampus, and amygdala) and a temporal lobe meta-ROI (bilateral parahippocampal gyrus, fusiform gyrus, inferior and middle temporal gyrus, and amygdala) [53].

### Statistical analyses

Between-group comparisons were used to describe both samples. We examined amyloid PET positivity group differences in the HOMA-IR sample and diabetic status group differences in the Diabetic Status sample. In the latter sample, we also determined diabetic status group differences within amyloid PET positive and negative groups. Between-group differences were tested using independent samples *t*-test for continuous variables and Pearson chi-square for categorical variables with > 5 cases per cell, Fisher’s Exact test for variables with 2 categories and ≤ 5 cases in a cell, and Fisher-Freeman-Hamilton Exact test for variables with > 2 categories and ≤ 5 cases in a cell. For variables with > 2 categories, post hoc pairwise comparisons for significant main effects were conducted using a *z*-test for independent proportions and Benjamini-Hochberg correction to adjust for multiple comparisons.

Two separate multiple linear regression models tested the relationship of HOMA2-IR and diabetic status to EC tau PET SUVR. Two separate moderated regression models tested if the relationship of IR and diabetic status to EC tau PET SUVR was moderated by amyloid burden. Prior to calculating the 2 interaction terms (i.e., HOMA2-IR × amyloid PET DVR and diabetic status × amyloid PET DVR), amyloid PET DVR was centered at 1.19, the cut-off for amyloid PET positivity, and HOMA2-IR was centered at the sample mean. Age, sex, cognitive status, and amyloid PET DVR were controlled in all analyses. Cohort was additionally controlled in analyses performed on the Diabetic Status sample. Residual plots from each model were examined to evaluate assumptions of regression. If heteroscedasticity was detected, the Breusch-Pagan test was performed to confirm the presence of non-constant variance. For models demonstrating heteroscedasticity, we performed robust regression employing a heteroscedasticity-consistent standard error estimator (HC3) [54]. The same analysis plan was followed when testing secondary tau PET SUVR ROIs as dependent variables.

IBM® SPSS® version 27 and SAS® version 9.4 were utilized for statistical analyses.

## Results

**Fig. 1:**
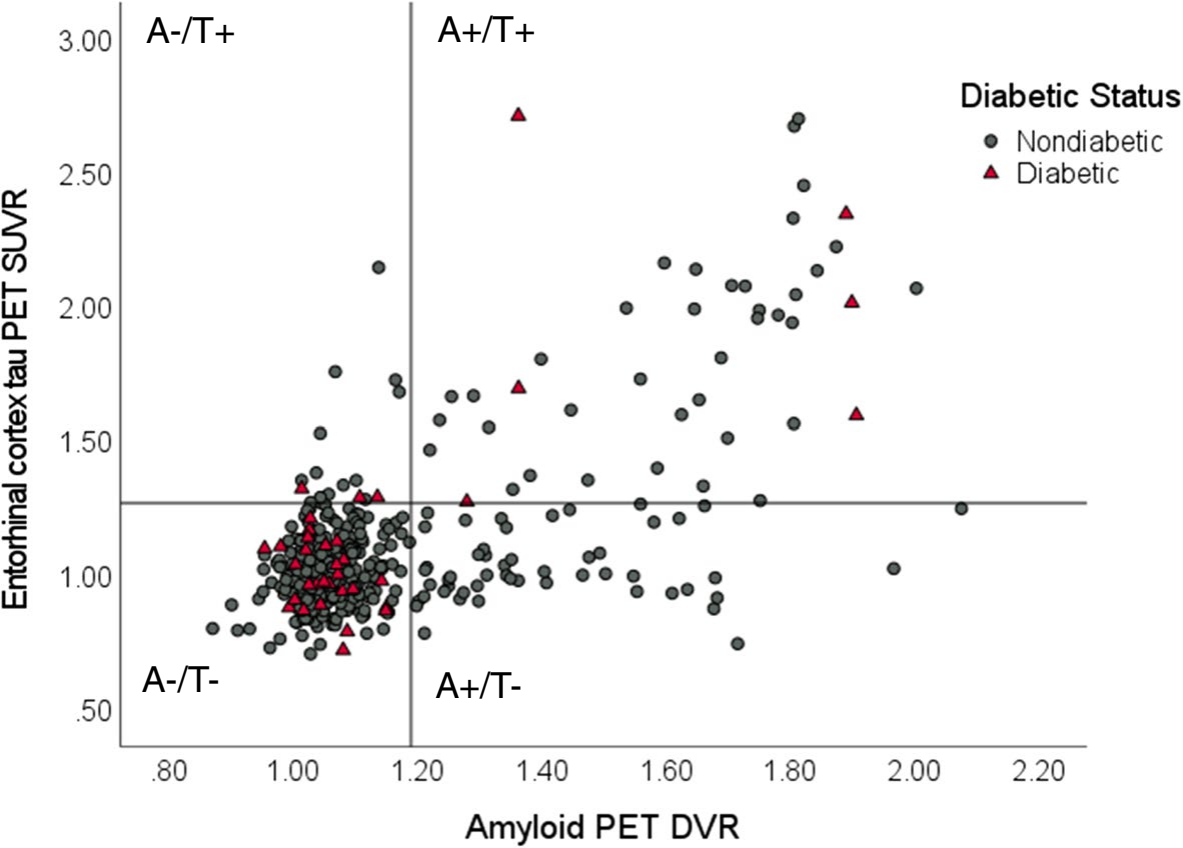
Scatterplot demonstrating relationship between amyloid PET DVR and entorhinal cortex tau PET SUVR in diabetic and nondiabetic participants in the Diabetic Status sample. Horizontal line set at tau PET positivity threshold (SUVR = 1.27); vertical line set at amyloid PET positivity threshold (DVR = 1.19). A, amyloid; T, tau

**Fig. 2:**
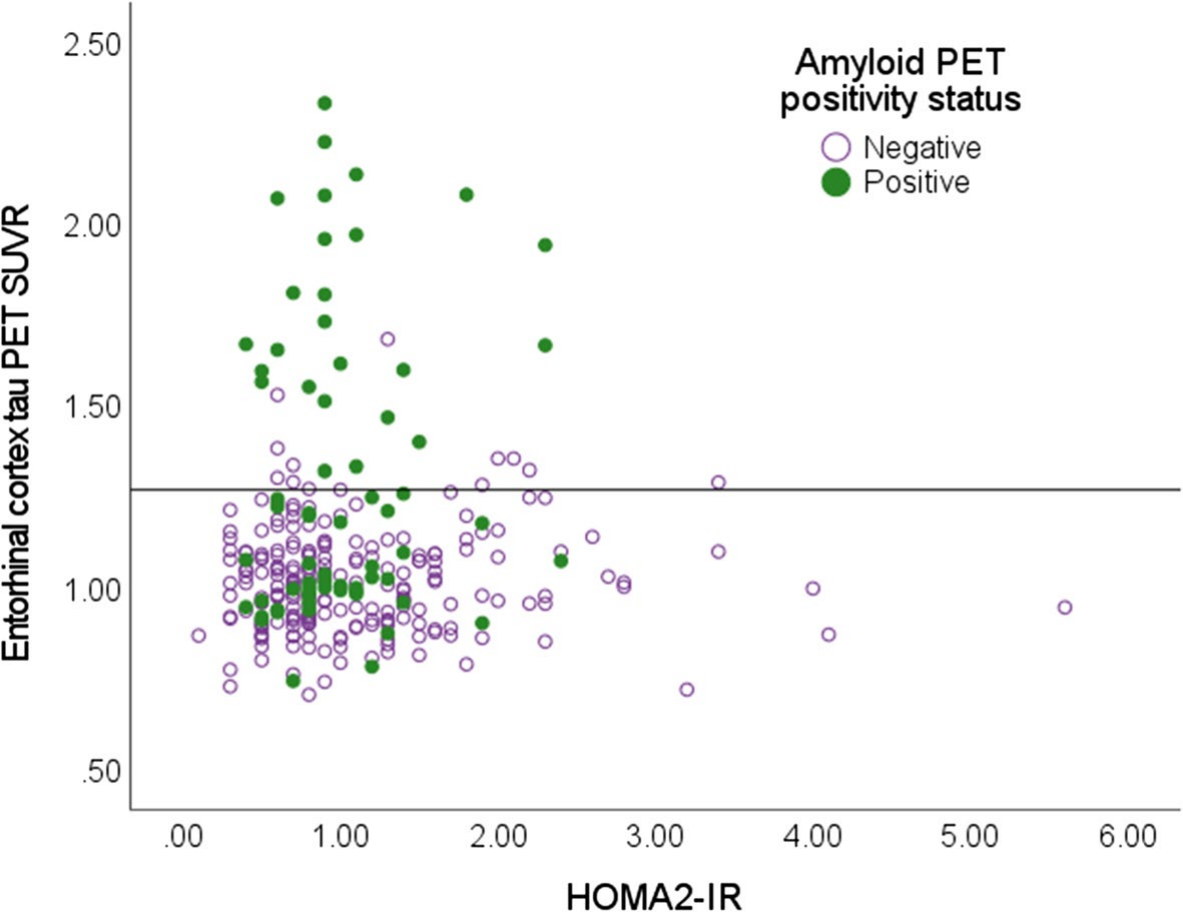
Scatterplot demonstrating relationship between HOMA2-IR and entorhinal cortex tau PET SUVR in amyloid PET positive and negative participants (threshold = 1.19 DVR). Horizontal line set at tau PET positivity threshold (SUVR = 1.27)

Between-group differences can be found in Tables 1 and 2. Antidiabetic medication usage by participants in the Diabetic Status sample can be found in Supplementary Table 1. In the HOMA-IR sample, HOMA2-IR was not significantly different between the amyloid PET positive (*n*=67) and negative (*n*=213) groups. In the Diabetic Status sample, amyloid PET DVR and the proportion of participants who were amyloid PET positive were not significantly different between the diabetic and nondiabetic groups. Among amyloid PET-positive participants, the diabetic group (*n*=6) had significantly higher EC and MTL tau PET SUVR than the nondiabetic group (*n*=88). Among amyloid PET-negative participants, EC and MTL tau PET SUVR was not significantly different between the diabetic (*n*=31) and nondiabetic (*n*=269) groups. A scatterplot demonstrating the relationship between amyloid PET DVR and EC tau PET SUVR in diabetic and nondiabetic participants in the Diabetic Status sample is presented in Fig. 1. A second scatterplot demonstrating the relationship between HOMA2-IR and EC tau PET SUVR is presented in Fig. 2.

Review of the residual plots revealed a likely violation of the homoscedasticity assumption in all 4 linear regression models. The Breusch-Pagan test was significant for each model (Supplementary Table 2), indicating the presence of non-constant variance. Thus, we ran robust regression employing a heteroscedasticity-consistent standard error estimator (HC3). Results from robust regression demonstrated that HOMA2-IR and diabetic status were not significantly related to EC tau PET SUVR (see Table 3). The HOMA2-IR × amyloid PET DVR and diabetic status × amyloid PET DVR interactions were also not significantly related to EC tau PET SUVR (see Table 3). When examining as dependent variables the secondary tau PET SUVR ROIs, we found that HOMA2-IR and diabetic status, as well as their interaction with amyloid PET DVR, were not significantly related to tau PET SUVR in the MTL and temporal lobe meta-ROIs (see Table 4). As in primary analyses, robust regression was used in these secondary analyses due to the presence of heteroscedasticity.

**Table 3:** Results from robust regression testing the relationships of a) HOMA2-IR and diabetic status, as well as b) their interaction with amyloid PET DVR, to tau PET SUVR in the entorhinal cortex^a^

	HOMA-IR sample			Diabetic Status sample		
	*b* (SE)^b^	*p*	95% CI	*b* (SE)^b^	*p*	95% CI
(a)						
Age (years)	.001 (.002)	.42	−.002 to .005	.004 (.002)	.02	.001 to .007
Sex (0=female)	−.06 (.03)	.04	−.11 to −.003	−.02 (.03)	.56	−.07 to .04
Cognitive status (0=unimpaired)^c^	.20 (.13)	.13	−.06 to .46	.22 (.09)	.02	.04 to .41
Amyloid PET DVR	.68 (.12)	<.001	.45 to .91	.79 (.10)	<.001	.60 to .99
HOMA2-IR	.02 (.02)	.23	−.01 to .05	--	--	--
Cohort (WRAP=0)	--	--	--	.08 (.03)	.004	.03 to .14
Diabetic status (0=nondiabetic)	--	--	--	.04 (.04)	.32	-.04 to .13
(b)						
Age (years)	.001 (.002)	.39	−.002 to .005	.004 (.002)	.04	.0002 to .007
Sex (0=female)	−.06 (.03)	.04	−.11 to −.003	−.03 (.03)	.30	−.08 to .03
Cognitive status (0=unimpaired)^c^	.19 (.13)	.16	.07 to .45	.23 (.09)	.01	.05 to .41
Amyloid PET DVR (centered)^d^	.69 (.13)	<.001	.44 to .93	.75 (.11)	<.001	.54 to .97
HOMA2-IR (centered)^e^	.03 (.03)	.42	−.03 to.09	--	--	--
HOMA2-IR × amyloid PET DVR	.10 (.23)	.68	−.36 to .55	--	--	--
Cohort (WRAP=0)	--	--	--	.08 (.03)	.008	.02 to .13
Diabetic status (0=nondiabetic)	--	--	--	.06 (.05)	.26	-.04 to .16
Diabetic status × amyloid PET DVR	--	--	--	.36 (.26)	.17	−.16 to .88

*Abbreviations*: *CI* Confidence interval; *DVR* Distribution volume ratio, *HOMA2-IR* Homeostasis model assessment of insulin resistance, *PET* Positron emission tomography, *SE* Standard error, *SUVR* standardized uptake value ratio

^a^Tau PET SUVR was the average value from bilateral entorhinal cortex

^b^Computed using a heteroscedasticity-consistent standard error estimator (HC3)

^c^*n*=9 with MCI in HOMA-IR sample; *n*=23 with MCI and *n*=6 with dementia in Diabetic Status sample

^d^Amyloid PET DVR centered at cut-off for amyloid PET positivity (DVR = 1.19)

^e^HOMA2-IR centered at mean

**Table 4:** Results from robust regression testing the relationship of HOMA2-IR and diabetic status, as well as their interaction with amyloid PET DVR, to tau PET SUVR in the medial temporal lobe and temporal lobe meta-ROI

	Tau PET SUVR, medial temporal lobe^a^			Tau PET SUVR, temporal meta-ROI^b^		
	*b* (SE)^c^	*p*	95% CI	*b* (SE)^c^	*p*	95% CI
HOMA2-IR^d^	.01 (.01)	.36	−.01 to .04	.004 (.01)	.68	−.02 to .03
HOMA2-IR × amyloid DVR^d^	.24 (.23)	.31	−.22 to .69	−.22 (.20)	.27	−.60 to .17
Diabetic status^e^ (0=nondiabetic)	.02 (.04)	.59	−.05 to .09	.01 (.03)	.69	−.05 to .07
Diabetic status × amyloid PET DVR^e^	.24 (.28)	.40	−.32 to .79	.22 (.25)	.37	−.26 to .71

*Abbreviations*: *CI* Confidence interval, *DVR* Distribution volume ratio, *HOMA2-IR* Homeostasis model assessment of insulin resistance, *PET* Positron emission tomography, *ROI* Region of interest, *SE* Standard error, *SUVR* Standardized uptake value ratio

^a^Average tau PET SUVR from bilateral entorhinal cortex, hippocampus, and amygdala

^b^Average tau PET SUVR from bilateral parahippocampal gyrus, amygdala, fusiform cortex, and inferior and middle temporal gyrus

^c^Computed using a heteroscedasticity-consistent standard error estimator (HC3)

^d^Analyses controlled for age, sex, and cognitive status. Amyloid PET DVR centered at cut-off for amyloid PET positivity (DVR = 1.19). HOMA2-IR centered at mean

^e^Analyses controlled for age, sex, cognitive status, and cohort. Amyloid PET DVR centered at cut-off for amyloid PET positivity (DVR = 1.19)

**Fig. 3:**
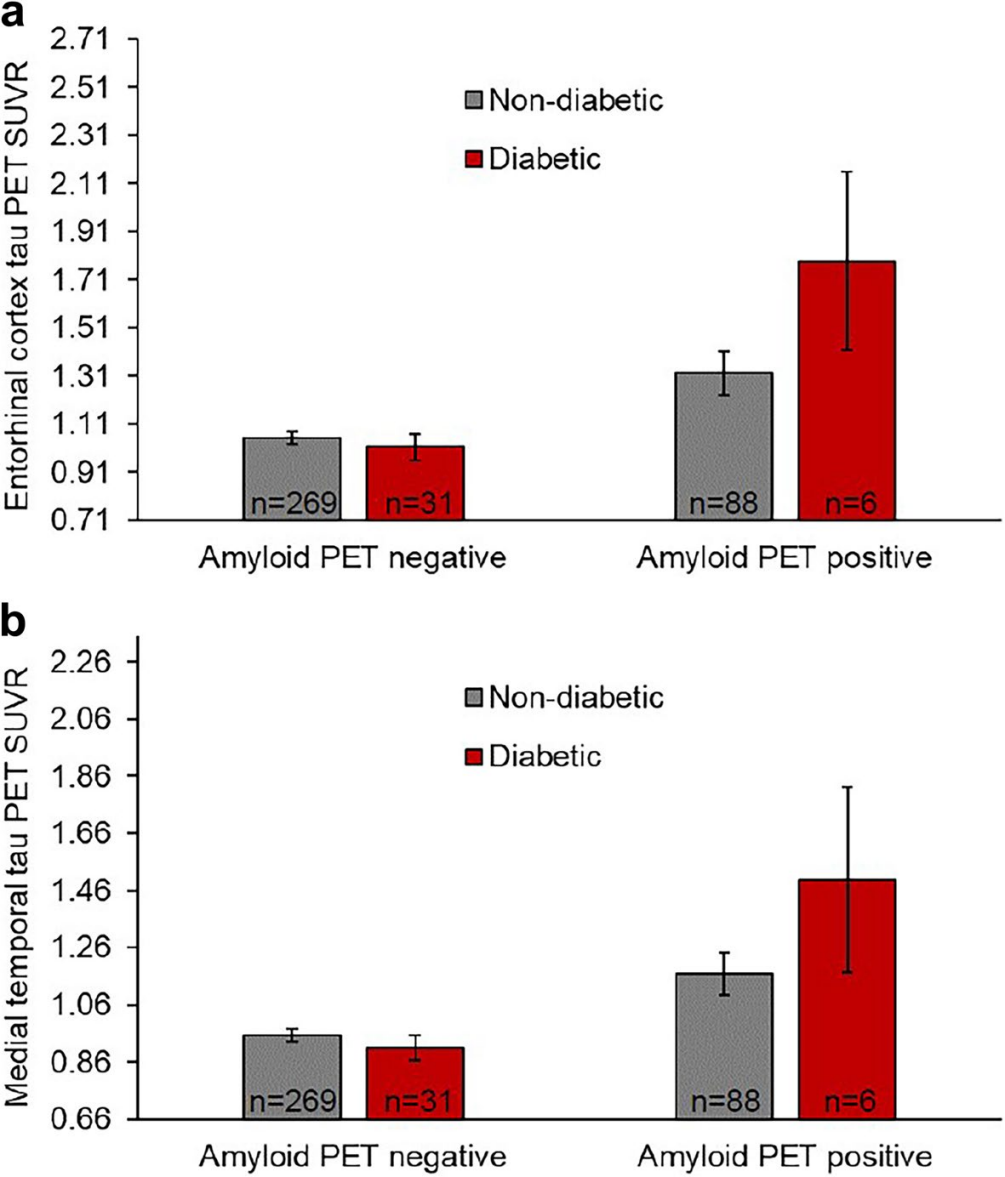
Estimated marginal means of **a** entorhinal cortex and **b** medial temporal lobe tau PET SUVR controlling for age, sex, cohort, and cognitive status. Medial temporal lobe tau PET SUVR = average tau PET SUVR from bilateral entorhinal cortex, hippocampus, and amygdala. Amyloid PET positivity threshold = 1.19 DVR. Error bars represent 95% confidence intervals

Because the diabetic amyloid PET positive group had higher EC and MTL tau PET SUVR than the nondiabetic amyloid PET positive group, we tested whether a diabetic status × amyloid PET positivity interaction would be significantly related to EC and MTL tau PET SUVR when controlling for age, sex, cohort, and cognitive status. Using robust regression with the HC3 estimator, we found that the interaction in both models was significant (EC tau PET SUVR model: *b* = .51, *p* = .01; MTL tau PET SUVR model: *b* = .37, *p* = .03; see Supplementary Table 3). To obtain EC and MTL tau PET SUVR estimated marginal means for each of the four diabetic status by amyloid PET positivity status groups, we used robust regression in SAS®. We then tested the difference in marginal means between the diabetic (EC tau PET SUVR mean = 1.79; MTL tau PET SUVR mean = 1.50) and nondiabetic (EC tau PET SUVR mean = 1.32 EC; MTL tau PET SUVR mean = 1.17) amyloid PET positive groups. We found that the diabetic amyloid PET positive group had a significantly higher mean EC tau PET SUVR than the nondiabetic amyloid PET positive group (*t* = 2.39, *p* = .02; see Fig. 3a). The difference in adjusted MTL tau PET SUVR means between the diabetic and nondiabetic amyloid PET positive groups was close to the significance threshold (*t* = 1.94, *p* = .05; see Fig. 3b). We then conducted a sensitivity analysis excluding participants with cognitive impairment (*n*=29) and controlling for age, sex, and cohort in a model testing EC tau PET SUVR as the outcome. The pattern of results was similar, but the diabetic status × amyloid PET positivity interaction (*b* = .33, *p* = .07) and the difference between the EC tau PET SUVR marginal means in the diabetic (mean = 1.56 SUVR, n=4) and nondiabetic (mean = 1.26 SUVR, *n*=70) amyloid PET positive groups was not significant (*t* = 1.70, *p* = .09). In additional robust regressions using the HC3 estimator and controlling for the same variables as in primary analyses, we found that a diabetic status × amyloid PET positivity interaction was not significantly related to tau PET SUVR in the temporal meta-ROI (*b* = .29, *p* = .09) and that an HOMA2-IR × amyloid PET positivity interaction was not significantly associated with EC tau PET SUVR (see Supplementary Table 3 for latter results).

## Discussion

HOMA2-IR was not significantly related to EC tau PET SUVR in a nondemented middle-aged and older adult sample enriched for AD risk. Additionally, the relationship between HOMA2-IR and EC tau PET SUVR was not moderated by amyloid PET DVR or amyloid PET positivity status. Similarly, diabetic status was not related to EC tau PET SUVR and the interaction between diabetic status and amyloid PET DVR was also not significantly associated with EC tau PET SUVR. HOMA2-IR and diabetic status, as well as their interaction with amyloid PET DVR, were also not related to tau PET SUVR in secondary ROIs, the MTL, and temporal meta-ROI. Despite the small number in the diabetic amyloid PET positive subset (*n*=6), a significant interaction between diabetic status and amyloid PET positivity status was related to EC and MTL tau PET SUVR. Being diabetic and amyloid PET positive was associated with higher EC and MTL tau PET SUVR. We discuss these findings in the context of current research examining relationships between IR, diabetes, and AD-related pathologic tau.

Our HOMA2-IR findings are congruent with the results of two previous post-mortem studies that found no significant relationship between antemortem HOMA1-IR and Braak score [25, 26]. Although other post-mortem studies [55–57] have described IR [57] or a reduction in the levels of insulin signaling kinases (e.g., PI3K) [55, 56] in the brain tissue of AD cases confirmed to have NFTs and amyloid plaques, it is not known from these studies whether peripheral IR contributed to brain IR in the AD cases observed. A measure of antemortem peripheral IR was not reported, and AD cases determined to have brain IR did not have a known antemortem type 2 diabetes diagnosis [57], suggesting that peripheral IR associated with type 2 diabetes was not essential for the development of brain IR. Whether or under what conditions peripheral IR found in prediabetes or type 2 diabetes is related to brain IR warrants investigation. Results from animal studies investigating a linkage between peripheral and central IR have been mixed [58–61].

Despite any linkage between peripheral and brain IR, it remains unclear if brain IR precedes or facilitates tau aggregation. Some have hypothesized that the facilitation of AD-related tau hyperphosphorylation occurs due to overactivity of glycogen synthase kinase-3 (GSK-3) [62], an insulin signaling kinase whose active form phosphorylates tau. However, in contrast to that hypothesis, post-mortem studies have not found higher total levels of GSK-3 in AD cases relative to controls [55, 57]. Furthermore, levels of the suppressed (i.e., inactive) form of GSK-3 were found to be positively (rather than negatively) related to NFT density [57]. Instead of causing tau aggregation, brain IR could be a consequence. In a cellular model, tau hyperphosphorylation specific for AD was shown to drive intracellular insulin aggregation and subsequent IR, demonstrating that abnormal tau in AD could precede brain IR [63]. Whether peripheral IR is related to brain IR in AD and has an upstream influence on the development of tau pathology requires further investigation.

In addition to causing metabolic dysfunction, IR and type 2 diabetes also have adverse effects upon the vascular endothelium [31, 32] and increase the risk for cardiovascular disease [33, 34]. Results from some studies suggest that a synergistic association between vascular disease risk and higher cortical amyloid is related to greater tau pathology [35, 36]. In contrast to these findings, we did not find that individuals with higher HOMA2-IR values and greater cortical amyloid burden, determined by amyloid PET DVR and positivity status, had higher tau PET SUVR in the EC and secondary ROIs. Results may have been limited by the small proportion (14.6%) of participants with HOMA2-IR greater than the mean value (1.6) of the diabetic participants.

Tau PET SUVR in the EC and secondary ROIs was also not related to an interaction between diabetic status and amyloid PET DVR; however, tau PET SUVR in the EC and MTL was significantly associated with an interaction between diabetic status and amyloid PET positivity status. The diabetic amyloid PET positive group had significantly higher EC tau PET SUVR than the non-diabetic amyloid PET positive group. The difference between mean MTL tau PET SUVR in the diabetic and nondiabetic amyloid PET positive groups was similar in direction but weaker statistically (*p* = .05). The diabetic × amyloid PET positivity interaction was not significantly related to tau PET SUVR in the temporal meta-ROI (*p* = .09). Since the EC is one of the first regions to develop NFTs according to Braak pathological staging [50], the difference in findings between the EC and secondary ROIs could have been due to greater tau accumulation in the EC and less extensive spread of tau throughout the MTL and temporal lobe. In general, the interpretation of the results is limited by the small number of participants who were both diabetic and amyloid PET positive (*n*=6). Participants who were cognitively impaired likely contributed to the significant association between the diabetic status × amyloid PET positivity interaction and EC tau PET SUVR. Following the removal of cognitively unimpaired participants (including 2 of the 6 with diabetes and amyloid PET positivity), that interaction was similar in direction but was no longer significant. It is also likely that the removal of participants from the sample reduced the statistical power to detect a significant effect. Findings suggest the need for additional study to examine if diabetes, through its diverse effects on the vasculature and cellular metabolism, predisposes neurons to tangle formation when amyloid reaches a certain level.

It is noteworthy that neuropathological studies do not support a relationship between type 2 diabetes and NFT or amyloid plaque pathology [27–30], suggesting that either diabetes is not an instigator of AD pathology or possibly that medications for type 2 diabetes help to mitigate AD pathogenesis. In contrast, diabetes has been consistently related to indicators of cerebrovascular disease, such as microinfarcts, lacunes, or white matter hyperintensities [30]. Nevertheless, population-based studies of large sample sizes regularly find that type 2 diabetes is associated with an increased risk for Alzheimer’s clinical syndrome [64–66]. Similar associations to increased Alzheimer’s clinical syndrome risk have been found for elevated HOMA-IR and hyperinsulinemia (an indicator of IR) [3, 4, 67]. Because the diagnosis of dementia in epidemiological studies has not been complemented for practical reasons by CSF or PET biomarkers of AD pathology, it is possible that some individuals with vascular dementia in these studies were misdiagnosed as having dementia due to AD or reflected the increased presentation of the AD clinical syndrome among individuals with mixed AD and vascular pathology [68]. Meta-analyses of epidemiologic studies have indicated that people with type 2 diabetes have a greater risk for vascular than AD dementia; however, when accounting for cerebrovascular and cardiovascular disease, the risk for clinical AD, although reduced, is still present [64].

Mechanisms that may account for the relationship between type 2 diabetes and Alzheimer’s clinical syndrome are unclear [69]. Whether diabetes interacts with amyloid to influence cognitive decline in AD should be examined. Some studies support a synergistic effect between AD biomarkers and vascular risk or vascular endothelial dysfunction upon cognitive function and decline [35, 70]; however, others provide evidence for additive effects, indicating that vascular risk factors and AD pathology promote cognitive decline through independent pathways [71, 72]. Differences in results could be due to differences in sample composition, especially in the proportion of participants effectively treated to reduce vascular risk. Participants untreated for vascular disease risk factors have been shown to have more AD pathology than treated counterparts [73, 74]; thus, identifying a synergistic association between vascular risk and AD pathology upon cognitive decline may be more likely in samples that contain individuals who have not been treated for vascular disease risk factors.

Our study has several strengths and limitations. The use of the tau PET radioligand ^18^F-MK-6240 to detect aggregated tau was a strength of this study. ^18^F-MK-6240 is a second-generation tracer that has demonstrated high affinity and selectivity to AD-type NFTs comprised of PHF tau in post-mortem AD brains and little to no binding to tau aggregates in non-AD tauopathy [75]. Our samples were enriched for AD risk due to increased proportion of *APOE* ε4 allele carriers, and 23.9% of participants in each sample had a positive amyloid PET. There were no participants in the HOMA-IR sample with dementia, and only 1.5% in the Diabetic Status sample had been diagnosed with dementia. This allowed us to examine the relationship of IR and diabetic status to tau PET SUVR in a sample containing individuals who were in the early stages of the AD clinicopathologic continuum, thus extending results from previous studies that investigated the relationship of IR or diabetic status to NFTs in the post-mortem brain tissue of older adults with and without an antemortem dementia diagnosis [26–28].

Because of the correlational nature of our study, causative claims cannot be made. Because of the small number of participants who were diabetic, we did not have adequate power to detect small effects of diabetic status on tau PET SUVR.

Results should be interpreted within the context of sample characteristics. Both samples were comprised predominantly of well-educated and relatively healthy participants. The proportion of participants with diabetes was lower than the population prevalence in Wisconsin for older adults. There were 7.9% in the HOMA-IR and 9.4% in the Diabetic Status sample who were identified as diabetic. These values are slightly less than the 10.9% prevalence rate for diabetes in middle-aged (45–64 years) adults and lower than the 18.3% and 20.6% prevalence rates, respectively for youngest old (65–74 years) and mid to oldest old adults (> 75 years) in Wisconsin in the year 2018 [76]. It is possible that some participants with undiagnosed diabetes were missed due to the lack of 2-hour post-load glucose or hemoglobin A1c measurements.

Because most diabetics (73.0%) in the Diabetic Status sample reported taking antidiabetic medication, we were unable to investigate if untreated diabetes was related to tau. Although a post-mortem study did not find any significant differences in NFT pathology between treated and untreated diabetics [77], another study of living participants found that the amount of CSF P-tau181 in treated diabetic, prediabetic and nondiabetic groups was similar and significantly lower than the concentration in a group of untreated diabetics [74]. Oral antidiabetic medication has been shown in some but not all studies to reduce the risk of dementia [64]. Whether untreated diabetes and the exacerbation of associated conditions, such as inflammation and oxidative stress, play a role in the development of tau pathology deserve investigation.

Relatedly, we acknowledge that effective blood glucose control and diabetes duration could have influenced results. Longer diabetes duration was related in one study to elevated CSF P-tau-181 [74]. Since we did not have hemoglobin A1c to assess glucose control or data on diabetes duration, we were unable to study whether these factors were related to tau PET SUVR. Research is needed to investigate not only untreated diabetes but also the extent that glucose control and diabetes duration are related to AD-associated hyperphosphorylated and aggregated tau.

The majority (90.9% to 93.9%) of participants in our samples self-identified as White. African Americans, American Indians, and some Hispanic and Asian American ethnic groups have higher rates of diabetes than non-Hispanic White Americans [78, 79]. Although there was a greater proportion of African Americans and other minoritized groups in the diabetic relative to the non-diabetic group in our sample, there were insufficient numbers for stratified analyses. Studies with more diverse participant representation are needed.

In conclusion, HOMA2-IR was not associated with EC tau PET SUVR in a sample enriched for AD risk and comprised predominantly of middle-aged and older adults who were cognitively unimpaired. This finding is in congruence with results from previous post-mortem studies [25, 26] and suggests that IR may not be related to the early presence of tau aggregates in AD. Replication is needed in a sample representing a diversity of racial and ethnic groups and with a larger proportion of diabetic participants. Findings for diabetic status were inconclusive but suggest the need for studies investigating whether a synergistic association between diabetes and amyloid is related to tau. Future studies should also examine the role of diabetic treatment effectiveness and diabetes duration upon the development of tau.

## Supplementary Information

**Additional file 1: Supplementary Table 1.** Antidiabetic medication usage in the Diabetic Status sample. Data presented are counts (%). **Supplementary Table 2.** Results from linear regression testing (a) the relationship of IR and diabetic status to entorhinal cortex tau PET SUVR^1^ and (b) amyloid PET DVR as a moderator of the relationship of HOMA2-IR and diabetic status to entorhinal cortex tau PET SUVR. **Supplementary Table 3.** Results from robust regression testing amyloid PET positivity status as a moderator of the relationship of diabetic status and HOMA2-IR to tau PET SUVR in the a) entorhinal cortex^1^ and b) medial temporal lobe^2^.

## Data Availability

Data may be requested by making application for it from WRAP (https://wrap.wisc.edu/data-requests/) and WI-ADRC (https://www.adrc.wisc.edu/apply-resources). Please reference this journal article when making your request.

## Abbreviations

AD: Alzheimer’s disease
APOE: Apolipoprotein E
CSF: Cerebrospinal fluid
CT: Computed tomography
DVR: Distribution volume ratio
EC: Entorhinal cortex
GSK: Glycogen synthase kinase
HOMA2-IR: Homeostasis model assessment of insulin resistance
IR: Insulin resistance
MCI: Mild cognitive impairment
MTL: Medial temporal lobe
MRI: Magnetic resonance imaging
NFT: Neurofibrillary tangle
PHF: Paired helical filament
PET: Positron emission tomography
PiB: ^11^C-Pittsburgh Compound B
ROI: Region of interest
SUVR: Standardized uptake value ratio
WI-ADRC: Wisconsin Alzheimer’s Disease Research Center
WRAP: Wisconsin Registry for Alzheimer’s Prevention
