# Supplementary material for: The relationship of insulin resistance and diabetes to tau PET SUVR in middle-aged to older adults
Source: Alzheimers Res Ther. 2023 Mar 17;15:55. doi: 10.1186/s13195-023-01180-2 (PMC10022314; doi:10.1186/s13195-023-01180-2)
Supplement: Supplementary file 1 — Additional file 1: Supplementary Table 1. Antidiabetic medication usage in the Diabetic Status sample. Data presented are counts (%). Supplementary Table 2. Results from linear regression testing (a) the relationship of IR and diabetic status to entorhinal cortex tau PET SUVR1 and (b) amyloid PET DVR as a moderator of the relationship of HOMA2-IR and diabetic status to entorhinal cortex tau PET SUVR. Supplementary Table 3. Results from robust regression testing amyloid PET positivity status as a moderator of the relationship of diabetic status and HOMA2-IR to tau PET SUVR in the a) entorhinal cortex1 and b) medial temporal lobe2. [file 13195_2023_1180_MOESM1_ESM.docx]

Supplementary Table 1. Antidiabetic medication usage in the Diabetic Status sample. Data presented are counts (%).

|  | Diabetic Status  (Sample size = 394) | Diabetic amyloid PET positive  (Subsample size=6) | Non-diabetic amyloid PET positive  (Subsample size=88) | Diabetic amyloid PET negative  (Subsample size=31) | Non-diabetic amyloid PET negative  (Subsample size=269) |
| --- | --- | --- | --- | --- | --- |
| *Usage of any antidiabetic medication*  Diabetic  Nondiabetic^1^ | 27 (6.9)  4 (1.0) | 3 (50.0)  -- | --  0 (0.0) | 24 (77.4)  -- | --  4 (1.5) |
| *Antidiabetic medication class used* | | | | | |
| Metformin only | 23 (5.8) | 3 (50.0) | 0 (0.0) | 16 (51.6) | 4 (1.5) |
| Sulfonylurea only | 2 (0.5) | 0 (0.0) | -- | 2 (6.5) | -- |
| Metformin + sulfonylurea | 2 (0.5) | 0 (0.0) | -- | 2 (6.5) | -- |
| Metformin + sulfonylurea + sodium-glucose co-transporter-2 inhibitor | 1 (0.25) | 0 (0.0) | -- | 1 (3.2) | -- |
| Metformin + sulfonylurea + glucagon-like peptide-1 receptor agonist | 1 (0.25) | 0 (0.0) | -- | 1 (3.2) | -- |
| Metformin + dipeptidyl peptidase-4 inhibitor + insulin | 1 (0.25) | 0 (0.0) | -- | 1 (3.2) | -- |
| Sulfonylurea + glucagon-like peptide-1 receptor agonist + insulin | 1 (0.25) | 0 (0.0) | -- | 1 (3.2) | -- |

^1^ Off-label use of metformin in non-diabetics.

Supplementary Table 2. Results from linear regression testing (a) the relationship of IR and diabetic status to entorhinal cortex tau PET SUVR^1^ and (b) amyloid PET DVR as a moderator of the relationship of HOMA2-IR and diabetic status to entorhinal cortex tau PET SUVR.

(a)

|  | HOMA-IR sample | | | Diabetic status sample | | |
| --- | --- | --- | --- | --- | --- | --- |
|  | *b* (SE) | *p* | 95% CI | *b* (SE) | *p* | 95% CI |
| Age (years) | .001 (.002) | .49 | -.002 to .005 | .004 (.002) | .04 | .0001 to.007 |
| Sex (0 = female) | -.06 (.03) | .03 | -.11 to -.005 | -.03 (.03) | .29 | -.08 to .02 |
| Cognitive status (0=unimpaired)^2^ | .20 (.07) | .007 | .06 to .34 | .22 (.05) | <.001 | .12 to .33 |
| Amyloid PET DVR | .68 (.06) | <.001 | .56 to .79 | .79 (.06) | <.001 | .68 to .91 |
| HOMA2-IR | .02 (.02) | .34 | -.02 to .05 | -- | -- | -- |
| Cohort (WRAP=0) | -- | -- | -- | .08 (.03) | .004 | .03 to .14 |
| Diabetic status (0=nondiabetic) | -- | -- | -- | .04 (.04) | .29 | -.04 to .13 |
| Bruesch-Pagan Test | χ^2^(5) = 187.5, *p* < .001 | | | χ^2^(6) = 285.0, *p* < .001 | | |

(b)

|  | HOMA-IR sample | | | Diabetic Status sample | | |
| --- | --- | --- | --- | --- | --- | --- |
|  | *b* (SE) | *p* | 95% CI | *b* (SE) | *p* | 95% CI |
| Age (years) | .001 (.002) | .46 | -.002 to .005 | .004 (.002) | .045 | .00009 to .007 |
| Sex (0=female) | -.06 (.03) | .03 | -.11 to -.005 | -.03 (.03) | .28 | -.08 to .02 |
| Cognitive status (0=unimpaired)^2^ | .19 (.07) | .01 | .04 to .34 | .23 (.05) | <.001 | .13 to .33 |
| Amyloid PET DVR (centered)^3^ | .69 (.06) | <.001 | .57 to .80 | .75 (.06) | <.001 | .63 to .87 |
| HOMA2-IR (centered)^4^ | .03 (.02) | .21 | -.02 to .07 | -- | -- | -- |
| HOMA2-IR x Amyloid PET DVR | .10 (.12) | .43 | -.14 to .33 | -- | -- | -- |
| Cohort (0=WRAP) | -- | -- | -- | .08 (.03) | .007 | .02 to .13 |
| Diabetic status (0=nondiabetic) | -- | -- | -- | .06 (.04) | .16 | -.02 to .14 |
| Diabetic status x Amyloid PET DVR | -- | -- | -- | .36 (.17) | .04 | .02 to .69 |
| Bruesch-Pagan Test | χ^2^(6) = 190.3, *p* < .001 | | | χ^2^(7) = 289.4, *p* < .001 | | |

Abbreviations: CI = confidence interval; DVR = distribution volume ratio; HOMA2-IR = homeostasis model assessment of insulin resistance; PET = positron emission tomography; SE = standard error; SUVR = standardized uptake value ratio

^1^ Average tau PET SUVR from bilateral entorhinal cortex.

^2^ n=9 with MCI in HOMA-IR sample; n=23 with MCI and n=6 with dementia in Diabetic Status sample.

^3^ Amyloid PET DVR centered at cut-off for amyloid PET positivity (DVR = 1.19).

^4^ HOMA2-IR centered at mean.

Supplementary Table 3. Results from robust regression testing amyloid PET positivity status as a moderator of the relationship of diabetic status and HOMA2-IR to tau PET SUVR in the a) entorhinal cortex^1^ and b) medial temporal lobe^2^.

1. Tau PET SUVR, entorhinal cortex

|  | Diabetic Status sample | | | HOMA-IR sample | | |
| --- | --- | --- | --- | --- | --- | --- |
|  | *b* (SE)^3^ | *p* | 95% CI | *b* (SE)^3^ | *p* | 95% CI |
| Age (years) | .005 (.002) | .01 | .001 to .008 | .002 (.002) | .18 | -.001 to .006 |
| Sex (0=female) | -.04 (.03) | .12 | -.10 to .01 | -.07 (.03) | .03 | -.13 to -.007 |
| Cognitive status (0=unimpaired)^4^ | .33 (.10) | .001 | .13 to .52 | .26 (.16) | .10 | -.05 to .57 |
| Amyloid PET positivity (0=negative) | .27 (.05) | <.001 | .17 to .36 | .26 (.05) | <.001 | .15 to .37 |
| Cohort (WRAP = 0) | .07 (.03) | .04 | .005 to .13 | -- | -- | -- |
| Diabetic status (0=nondiabetic) | -.04 (.03) | .20 | -.10 to .02 | -- | -- | -- |
| Diabetic status x Amyloid PET positivity status | .51 (.20) | .01 | .12 to .89 | -- | -- | -- |
| HOMA2-IR^5^ |  |  |  | .01 (.01) | .44 | -.02 to .04 |
| HOMA2-IR x Amyloid PET positivity status |  |  |  | .05 (.12) | .68 | -.18 to .28 |

1. Tau PET SUVR, medial temporal lobe

|  | Diabetic Status sample | | | HOMA-IR sample | | |
| --- | --- | --- | --- | --- | --- | --- |
|  | *b* (SE)^3^ | *p* | 95% CI | *b* (SE)^3^ | *p* | 95% CI |
| Age (years) | .003 (.001) | .06 | -.0002 to .005 | .002 (.001) | .28 | -.001 to .005 |
| Sex (0=female) | -.02 (.02) | .47 | -.06 to .03 | -.04 (.03) | .13 | -.09 to .01 |
| Cognitive status (0=unimpaired)^4^ | .29 (.09) | .001 | .12 to .47 | .20 (.14) | .13 | -.06 to .47 |
| Amyloid PET positivity (0=negative) | .22 (.04) | <.001 | .14 to .29 | .21 (.05) | <.001 | .11 to .30 |
| Cohort (WRAP = 0) | .05 (.03) | .04 | .002 to .10 | -- | -- | -- |
| Diabetic status (0=nondiabetic) | -.04 (.03) | .09 | -.09 to .006 | -- | -- | -- |
| Diabetic status x Amyloid PET positivity status | .37 (.17) | .03 | .04 to .71 | -- | -- | -- |
| HOMA2-IR^5^ |  |  |  | .001 (.01) | .95 | -.02 to .02 |
| HOMA2-IR x Amyloid PET positivity status |  |  |  | .09 (.12) | .43 | -.14 to .32 |

Abbreviations: CI = confidence interval; HOMA2-IR = homeostasis model assessment of insulin resistance; PET = positron emission tomography; SE = standard error; SUVR = standardized uptake value ratio

^1^ Average tau PET SUVR from bilateral entorhinal cortex.

^2^ Average tau PET SUVR from bilateral entorhinal cortex, hippocampus, and amygdala.

^3^ Computed using a heteroscedasticity-consistent standard error estimator (HC3).

^4^ n=23 with MCI and n=6 with dementia in Diabetic Status sample; n=9 with MCI in HOMA-IR sample.

^5^ HOMA2-IR centered at mean.
